# Color variability and body size of larvae of two *Epomis* species (Coleoptera, Carabidae) in Israel, with a key to the larval stages

**DOI:** 10.3897/zookeys.119.1451

**Published:** 2011-07-15

**Authors:** Gil Wizen, Avital Gasith

**Affiliations:** Tel Aviv University, George S. Wise Faculty of Life Sciences, Department of Zoology, The National Collections of Natural History, Tel-Aviv 69978, Israel

**Keywords:** *Epomis* larvae, Carabidae, color atlas, body size

## Abstract

Species identification using the characteristics of developmental stages is challenging. However, for insect taxonomy the coloration of larval stages can be an informative feature. The use of live specimens is recommended for this because the color fades in preserved specimens. In this study we examine the possibility of using variation in coloration and color pattern of larvae in order to distinguish between twoground beetlesspecies *Epomis dejeani* (Dejean, 1831) and *Epomis circumscriptus* (Duftschmid, 1812). We present an atlas and describe the coloration and body size of the three larval stages of the above species based on live specimens. An identification key is given for the three larval instars of the two *Epomis* species.

The first instar larvae of the two *Epomis* species can be easily distinguished based on their color. From the second instar on, the variability in coloration and color patterns increases, creating an overlap in these attributes between larvae of the two species. Except for minor differences in color of the antennae and the base of the mandibles, larvae of the two species are indistinguishable at the second and third larval stages. To the best of our knowledge this is the first attempt to use variation in coloration and color pattern in live larvae in order to identify coleopterans. The color atlas of the larvae enables simple separation of the two *Epomis* species without requiring sophisticated magnifying devices, although it is less straightforward at the second and third larval stages.

We found similar body lengths between the two species for all developmental stages, except for third instar larvae prior to pupation. In the two species the difference in larval body length before pupation positively correlated with that of the adult beetles. More than 70% of the adults’ length can be explained by the length of the late third-instar larva; i.e. the large larvae develop into large adults. The larger specimens are the females.

## Introduction

Coloration can be an informative feature for insect taxonomy (van Emden 1957, Luff 1993). While the larvae of some ground beetles (Carabidae) have been well studied (reviewed in Lawrence 1991) for other beetles the larval stage is still unknown. Those larvae that have been studied were described according to morphology and structure, with less attention paid to color (e.g. van Emden 1942, Thompson 1979). Of the latter descriptions, some give limited information about the color of ground beetle larvae (e.g. van Emden 1942, Thompson 1979, Luff 1993, Erwin and Medina 2003), but not on pattern and color variability. Information available so far indicates that generally color variability in Coleoptera larvae is rare and so is the case in ground beetle larvae as well (Luff 1993, senior author personal observations).

In a recent study of two Chlaeniini (Carabidae) species of *Epomis*, *Epomis dejeani* (Dejean, 1831) and *Epomis circumscriptus* (Duftschmid, 1812), in Israel, we noticed that the larvae display color variation. The genus *Epomis* Bonelli, 1810 consists in ca. 20 species, mostly known from tropical Africa and south and south eastern Asia. Five of the species are from the Palaearctic region (Kryzhanovskij 1983). In mediterranean lands in Europe, this genus is rare and considered endangered (Brandmayr and Algieri 2000). *Epomis* have three larval stages (Elron et al. 2007). The third instar larva of *Epomis dejeani* was first described by Makarova (2005). Recently, Brandmayr et al. (2010) added a description of the first instar of *Epomis dejeani* and of the first and third instar larvae of *Epomis circumscriptus*. In none of the above morphological descriptions, however, is color variation in the larvae mentioned. This may be partly attributed to examination of larvae preserved in formaldehyde and alcohol. Preservation results in fading of the coloration of soft-bodied insects, particularly of the immature stages (McFarland 1964, Moore 1971, Luff 1993).

The taxonomic status of *Epomis* is under debate. Kirschenhofer (2003) considers *Epomis* as a subgenus of *Chlaenius*, whereas Basilewsky (1955) and Makarova (2005) consider it as a separate genus. Brandmayr et al. (2010), who examined and described the larvae of the two discussed species, support the separate standing of *Epomis* as a genus. Here we adopt the latter taxonomic approach.

We describe the variation in coloration and color pattern and body size of the three larval stages of *Epomis dejeani* and *Epomis circumscriptus*.

## Methods

The three larval stages of *Epomis* are referred to as L1, L2 and L3. Larvae were obtained *ex-ovo* in the laboratory from a dozen females of each species collected in the wild. The two *Epomis* species do not coexist at the same sites. Adults of each species were collected in a different locality from the largest population known for each species. The larvae were reared in a room with constant temperature (25oC ± 1oC) and artificial light. They were kept in one liter plastic containers (10.5 cm high; 14.5 cm diameter) with moist peat as substrate and were fed with live amphibian metamorphs. Freshly hatched or molted larvae appear uniformly white; the final color appears after about two hours. For this reason we used larvae 10 hours after hatching or 10 hours post-molt for documenting color variability and patterns. We photographed them using a Canon EOS 50D camera with Canon MP-E 65 mm and EF-S 60 mm macro lenses and Canon MT-24EX flash. We also measured larval body length from the tip of the mandible to the end of the abdomen (mandible-abdomen length, MAL) using a caliper (±0.05 mm). L1 was measured after hatching and before molting into L2; L2 was measured after molting and before molting into L3; and L3 was measured after molting and before pupation. We measured the body length of the adults that emerged (from the tip of the mandible to the end of the elytra), and examined the correlation between their body length with that of the late L3.

The statistical analysis was performed using Statistica ver. 8. Due to a small sample size and non-normal distribution of the data we used non-parametric statistical tests (Zar 1998). For comparing body lengths of each larval instar between the two species we used Mann-Whitney test. For analyzing the relationship of pre-pupation, third instar body length with the respective body length of the emerged adult we used Spearman correlation.

We prepared a key to the larvae, incorporating morphological characters described by Brandmayr et al. (2010) with the color characteristics and body length information recorded in this study. The description by Brandmayer et al. is based to a large degree on larvae we provided the senior author. Those larvae were from the same populations described above and were obtained in the same manner.

## Results

### Coloration and body length of E. dejeani larvae

**Figure 1.**
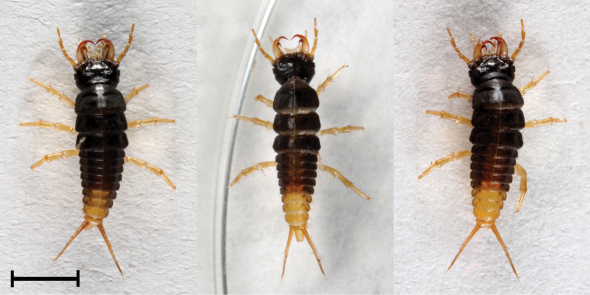
Morphs presenting color variability of L1 larvae of *Epomis dejeani*.Scale bar 2 mm.

**Figure 2.**
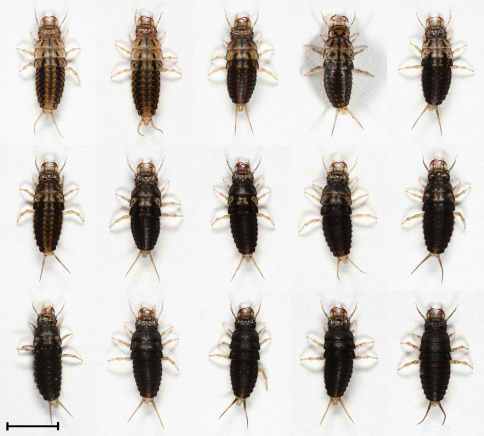
Morphs presenting color variability of L2 larvae of *Epomis dejeani*. Scale bar 5 mm.

**Figure 3.**
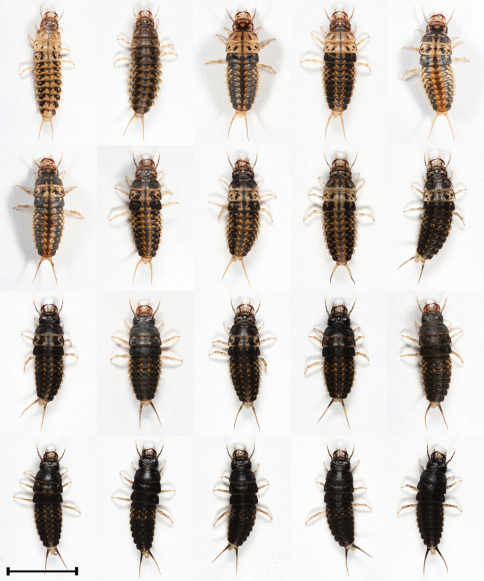
Morphs presenting color variability of L3 larvae of *Epomis dejeani*.Scale bar 10 mm.

L1, L2 and L3 differ in coloration. Moreover, we found variability in coloration within L2 and L3 stages (Fig. 2, 3 and 7). Median mandible-abdomen length (MAL) of L1 after hatching is 4.8 mm and before molting into L2 it is 8.3 mm (Table 1). The larva is mostly black-dark brown, with the last two or three segments of the abdomen being yellow. The tergite preceding the latter segments is medially yellow and its sides are black. The base of the mandible is pale brown or pale yellow. The retinaculum is reddish-brown and the apex is of similar but paler color. The remaining mouthparts, antennae, legs and urogomphi are pale yellow (Fig. 1). Median MAL of L2 after molting is 8.3 mm and before molting into L3 it is 13.2 mm (Table 1). The main body color of L2 ranges from pale brown with black spots to completely black. The legs and urogomphi are pale yellow and sometimes their base is black. The mandibles are pale brown or black, apex and retinaculum are reddish-brown. The base of the mandible is pale brown. The remaining mouthparts and antennae are pale brown or grey (Fig. 2 and 7). Median MAL of L3 after molting is 13.5 mm and before pupation it is 18.2 mm (Table 1). L3 resembles L2 in coloration, with the main body color ranging from pale brown with black spots to completely black. The legs are pale yellow. The urogomphi are mostly pale yellow but in dark colored larvae the base of the urogomphi is black. The mandibles are pale brown or black, apex and retinaculum are reddish-brown. The base of the mandible is pale brown. The remaining mouthparts and antennae are pale brown or grey (Fig. 3 and 7).

**Table 1.** Median body length (mm) of *Epomis dejeani* and *Epomis circumscriptus* larvae and inter-species MAL comparison (Mann-Whitney test) at different developmental stages. Number in parentheses indicates number of individuals.

Taxa	L1		L2		L3	
	After hatching	Prior to molting	After molting	Prior to molting	After molting	Prior to pupation
E. dejeani	4.8 (12)	8.3 (12)	8.3 (10)	13.2 (10)	13.5 (12)	18.2 (11)
E. circumscriptus	5 (11)	8.5 (9)	8.9 (10)	13 (9)	14 (19)	20.2 (14)
p-value	0.102	0.522	0.088	0.595	0.542	0.00084

### Coloration and body length of E. circumscriptus larvae

**Figure 4.**
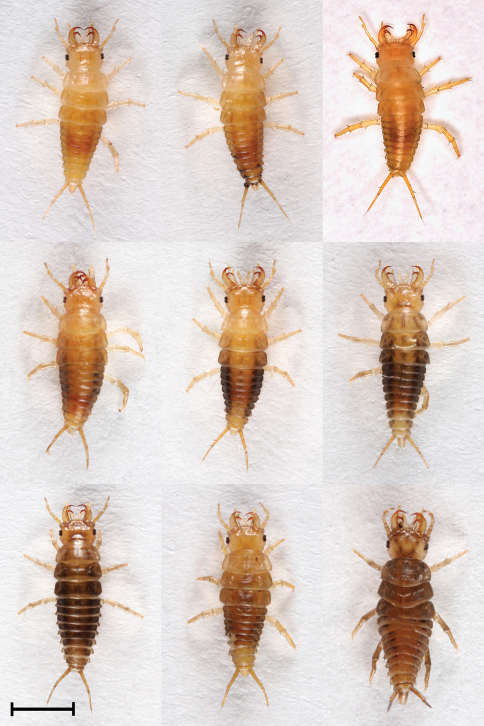
Morphs presenting color variability of L1 larvae of *Epomis circumscriptus*. Scale bar 2 mm.

**Figure 5.**
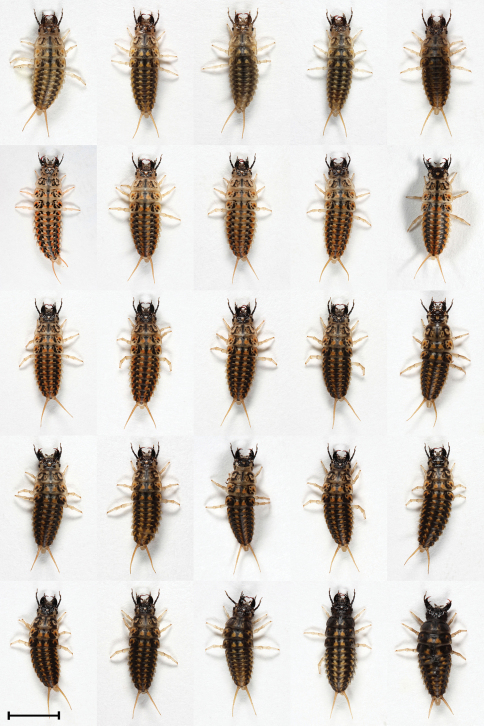
Morphs presenting color variability of L2 larvae of *Epomis circumscriptus*.Scale bar 5 mm.

**Figure 6.**
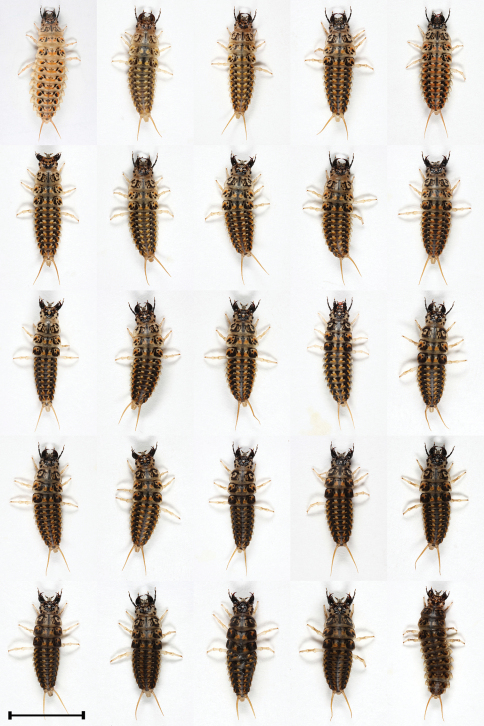
Morphs presenting color variability of L3 larvae of *Epomis circumscriptus*. Scale bar 10 mm.

**Figure 7.**
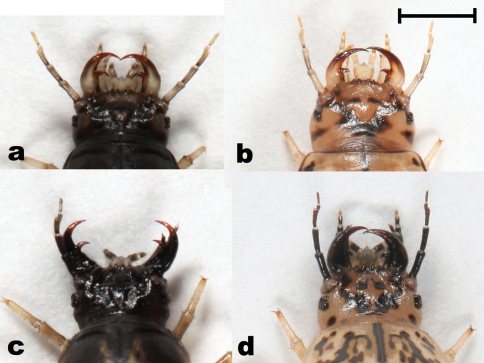
Head (dorsal view) of *Epomis* larvae showing differences in color of antennae and base of mandibles between the two species **a** *Epomis dejeani*, L2 dark morph **b** *Epomis dejeani*, L3 pale morph **c** *Epomis circumscriptus*, L2 dark morph **d** *Epomis circumscriptus*, L3 pale morph. Scale bar 2 mm.

L1, L2 and L3 differ in coloration. Here too we found variability in coloration within L2 and L3 stages (Fig. 5, 6 and 7). Median MAL of L1 after hatching is 5 mm and before molting into L2 it is 8.5 mm (Table 1). The larva L1 is mostly pale yellow or brownish and on rare occasions brown. In some larvae the sides of the body are grayish-black. The apex and retinaculum are reddish. The remaining mouthparts, antennae, legs and urogomphi are pale yellow. The dark eyes are prominent against the background of the brighter body (Fig. 4). Median MAL of L2 after molting is 8.9 mm and before molting into L3 it is 13 mm (Table 1). The main body color ranges from yellow-brown or white with black and orange spots to completely black with orange spots. The legs and urogomphi are yellow. The mandibles are black, apex and retinaculum are red. The remaining mouthparts are grey or black. The antennae are mostly black except for the two apical segments which are pale yellow (Fig. 5 and 7). Median MAL of L3 after molting is 14 mm and before pupation it is 20.2 mm (Table 1). The main body color ranges from dark brown or white with black and orange spots to completely black with orange spots. On rare occasions the orange spots are missing. The legs and urogomphi are yellow. The mandibles are black, apex and retinaculum are reddish-brown. The remaining mouthparts are grey or black. The antennae are mostly black except for the two last segments which are pale yellow (Fig. 6 and 7).

Except for L3 before pupation, the body length of L1, L2 and L3 of the two *Epomis* species is similar (Mann-Whitney, p>0.09, Table 1). Prior to pupation the body length of *Epomis circumscriptus* L3 is larger than that of *Epomis dejeani* at the same larval stage (Mann-Whitney, p<0.001; Table 1).

### Relationship between pre-pupal instar and adult body length

**Figure 8.**
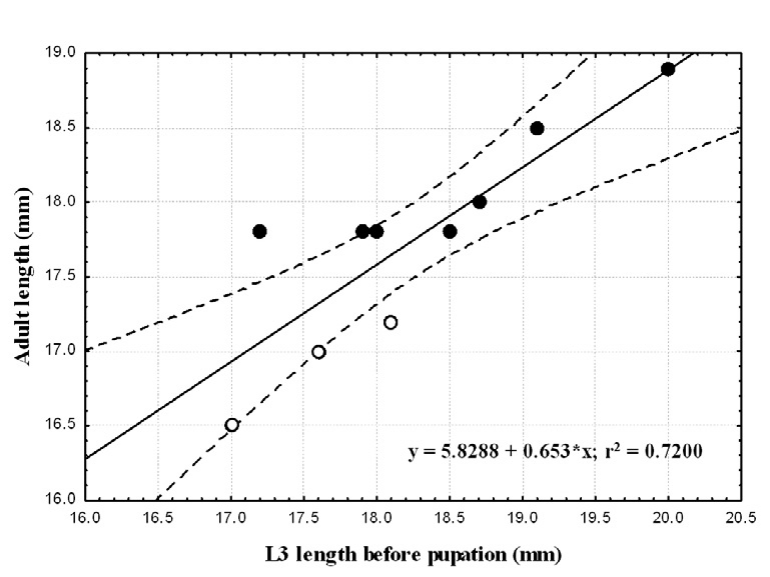
Correlation between adults’ body length (mm) and that of third-instar larvae (mm) of *Epomis dejeani* males (open circles) and females (black circles). Regression equation and coefficient of variation are shown.

**Figure 9.**
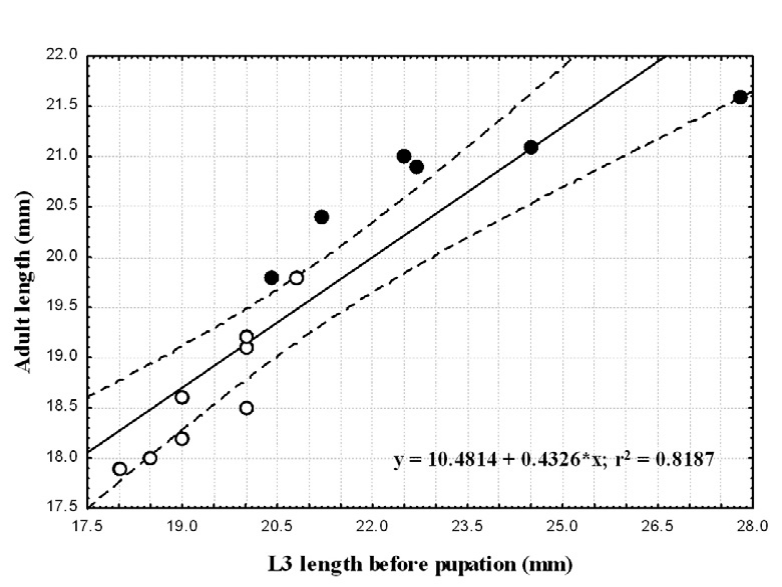
Correlation between adults’ body length (mm) and that of third-instar larvae (mm) of *Epomis circumscriptus* males (open circles) and females (black circles). Regression equation and coefficient of variation are shown.

For each species we compared body length of pre-pupal instar to that of the adult that emerged (Table 2). We found a positive significant correlation between larval and adult body length in each of the two species (Spearman correlation; p≤0.005). Larger larvae metamorphosed into larger adults. Larval size explains 72 and 82% of the variability in adult length in *Epomis dejeani* and *Epomis circumscriptus*, respectively (Table 2; Fig. 8 and 9). The largest larvae developed into females.

**Table 2.** Range and median of body length (mm) of third instar larvae before pupation and of adults (male and female) of *Epomis dejeani* and *Epomis circumscriptus*.

Taxa	Body length (mm)				Sex	Individual number
	L3 before pupation		Adult			
	range	median	range	median		
E. dejeani	17–18.1	17.6	16.5–17.2	17	M	3
	17.2–20	18.5	17.8–18.9	17.8	F	8
E. circumscriptus	18–20.8	19.5	17.9–19.8	18.6	M	8
	20.4–27.8	22.6	19.8–21.6	21	F	6

**Key to the larvae**

**Table:** 

1	Body length of the larva is up to 8.5 mm; egg bursters present on forehead, consisting of two rows of denticles; first instar	2
–	Body length of the larva is larger than 8.5 mm; no egg bursters present	3
2	Body length is 5 to 8.5 mm; color is mostly pale yellow or brownish, sometimes brown; body sides can sometimes be grayish-black; mandibles’ color is similar to body color, apex and retinaculum are darker and reddish; retinaculum on the same plane as the apical tooth and about the same length as the latter; both teeth strongly chitinized and curved, forming a strong double complanar hook; the remaining mouthparts, antennae, legs and urogomphi are pale yellow; stemmata are dark and clearly visible against the bright body color (Fig. 1)	*Epomis circumscriptus*
–	Body length is 4.8 to 8.3 mm; color is mostly black-dark brown, the last two or three segments of the abdomen are yellow, the tergite preceding these segments is medially yellow with black sides; mandible base is pale brown or pale yellow, retinaculum reddish-brown and apex of similar but paler color; retinaculum as robust as the apex, bent dorsally and backwards, not at the same plain as the apex, especially on the left side; both teeth strongly chitinized and curved, forming a strong double hook; the remaining mouthparts, antennae, legs and urogomphi are pale yellow; stemmata are poorly pigmented (Fig. 4)	*Epomis dejeani*
3	Body length of the larva is up to 13.5 mm; second instar	4
–	Body length of the larva is larger than 13.5 mm; third instar	5
4	Body length is 8.9 to 13.5 mm; color ranges from yellow-brown or white with black and orange spots to uniformly black with orange spots; the mandibles are black, apex and retinaculum are red; the remaining mouthparts are grey or black; the antennae are mostly black except for the two apical segments which are pale yellow; legs and urogomphi are yellow (Fig. 2 and 7)	*Epomis circumscriptus*
–	Body length is 8.3 to 13.2 mm; color ranges from pale brown with black spots to uniformly black; the mandibles are pale brown or black, mandible base is pale brown, apex and retinaculum are reddish-brown; the remaining mouthparts and antennae are pale brown or grey; legs and urogomphi are pale yellow, urogomphi sometimes black at base (Fig. 5 and 7)	*Epomis dejeani*
5	Body length is 14 to 20.2 mm; color ranges from dark brown or white with black and orange spots to uniformly black with or without orange spots; the mandibles are black, apex and retinaculum are reddish-brown; the remaining mouthparts are grey or black; the antennae are mostly black with the two last segments which are yellow; legs and urogomphi are yellow (Fig. 3 and 7)	*Epomis circumscriptus*
–	Body length is 13.5 to 18.2 mm; color ranges from pale brown with black spots to uniformly black; the mandibles are pale brown or black, mandible base is pale brown, apex and retinaculum are reddish-brown; the remaining mouthparts and antennae are pale brown or grey; legs pale yellow; urogomphi mostly pale yellow, and with a black base in uniformly black colored larvae (Fig. 6 and 7)	*Epomis dejeani*

## Discussion

*Epomis dejeani* and *Epomis circumscriptus* appear in the key to European Carabidae (Trautner and Geigenmüller 1987), which uses characters associated with identification of adults only. Makarova (2005) described for the first time the third-instar larva of *Epomis dejeani* (preserved specimen). We recently sent all three larval stages of the two *Epomis* species known from Israel to Pietro Brandmayr (University of Calabria, Italy) for description (Brandmayr et al. 2010). Brandmayr et al. reported a clear difference in mandible morphology in the first instar larvae of the two species. In *Epomis dejeani* larvae the retinaculum is bent dorsally while the apex is bent medially, whereas in *Epomis circumscriptus* larvae both retinaculum and apex are bent medially. A preliminary description of coloration of preserved larvae (in 70% alcohol) of the first and third larval stages is given. While studying the *Epomis* larvae we noticed that the color of preserved larvae fades with time, making color descriptions based on preserved specimens problematic. Here we present data on developmental changes reflected in variation in coloration and color pattern of the three larval stages, as well as body size. This is based on live specimens reared in the laboratory under the same conditions.

We found variability in color patterns in all larval stages of the two *Epomis* species. A couple of hours after hatching the first instar larvae of the two species can be easily distinguished based on their color. From the second instar on, the variability in coloration and color patterns increases, creating an overlap in these characteristics between larvae of the two species. Except for minor differences in color of the antennae and the base of the mandibles (Fig. 7), larvae of the two species are indistinguishable at the second and third larval stages. We found no significant difference in body length of larvae of the two *Epomis* species, except for the third instar larvae prior to pupation. At this stage the median body length of *Epomis circumscriptus* larvae is 2 mm longer than that of *Epomis dejeani* at the same stage. In the two species the difference in larval body length before pupation positively correlated with that of the adult beetles.

*Epomis* adults are the largest among the Chlaeniini (Basilewsky 1955). Here we found that in both *Epomis* species more than 70% of the adults’ length can be explained by the length of the late third instar larva; therefore, the large larvae develop into large adults. The larger specimens were the females. Although we have no additional evidence for a similar correlation between larval and adult body length in other carabids, this phenomenon is known from other Coleoptera (Stern and Emlen 1999).

According to the Catalogue of Palaearctic Coleoptera (Kirschenhofer 2003) and the key to the European Carabidae (Trautner and Geigenmüller 1987), *Epomis* is a subgenus of *Chlaenius*, and adults of *Epomis* are distinguished from their close *Chlaenius* relatives in their larger body length and the shape of their labial palp (Trautner and Geigenmüller 1987). Basilewsky (1955) examined the African fauna of *Epomis* and published a revision calling for separation of the genera. The description of the third instar larva of *Epomis dejeani* supports this conclusion (Makarova 2005). Brandmayr et al. (2010), who examined and described the larvae of the two species discussed in this study, also support the separate standing of *Epomis* as a genus within the Chlaeniini tribe. New data on the life history of the *Epomis* larvae (Wizen G and Gasith A, unpublished manuscript) indicate a unique biology of the *Epomis* beetles and therefore support the latter conclusion.

It should be pointed out that *Epomis* is an endangered taxon in the European Mediterranean region. *Epomis circumscriptus* was classified as a critically endangered species in Italy (Brandmayr and Algieri 2000). In Israel, *Epomis* populations are small and sparsely distributed. These findings and the fact that the larvae depends on a food source that is declining in many parts of the world (Alford and Richards 1999, Blaustein and Kiesecker 2002) suggests special attention for conservation measures that should be taken in order to protect these beetles from extinction.

In conclusion, the color atlas of *Epomis* larvae that we provide present the color pattern of yet little known larval stages of two ground beetle species, moreover it enables a simple separation of the two species without requiring sophisticated magnifying devices (for example ESM). The two species are easily distinguishable at the first larval stage. While the separation is less straightforward at the later stages, it is nonetheless possible by focusing on the color of the antennae and base of the mandibles. Accurate identification requires use of additional taxonomic tools. In another study (Settanni et al. 2009) the researchers used differential coloration of tergites in first instar larvae of two *Berberomeloe* species (Meloidae, Coleoptera) as diagnostic character for identification. However, their results rely on preserved specimens. To the best of our knowledge this is the first attempt to use variation in coloration and color pattern in live larvae in order to identify coleopterans.
